# Phospholipidomic Analysis Reveals Changes in Sphingomyelin and Lysophosphatidylcholine Profiles in Plasma from Patients with Neuroborreliosis

**DOI:** 10.1007/s11745-016-4212-3

**Published:** 2016-11-10

**Authors:** W. Łuczaj, P. Domingues, M. R. Domingues, S. Pancewicz, E. Skrzydlewska

**Affiliations:** 1 Department of Analytical Chemistry, Medical University of Bialystok, Bialystok, Poland; 2 Department of Chemistry, Mass Spectrometry Center, QOPNA, University of Aveiro, Aveiro, Portugal; 3 Department of Infectious Diseases and Neuroinfection, Medical University of Bialystok, Bialystok, Poland

**Keywords:** Phospholipids: Neuroborreliosis, Plasma, Mass spectrometry, Lipidomics

## Abstract

In recent years, the number of patients suffering from Lyme Disease (LD) has significantly increased. The most dangerous manifestation of LD is neuroborreliosis associated with invasion of the central nervous system by *Borrelia burgdorferi*. Phospholipids (PL) and their metabolites are involved in inflammation, which plays a dominant, but still unclear, role in the pathogenesis of neuroborreliosis. We analyzed the plasma PL profiles of neuroborreliosis patients (*n* = 8) and healthy volunteers (*n* = 8) using a lipidomic approach. Significant increases in the lysophosphatidylcholines LysoPtdCho 16:0 and LysoPtdCho 18:2 were observed. The plasma of neuroborreliosis patients appeared to have an increased relative abundance of sphingomyelin CerPCho d18:1/24:1 and a decrease in CerPCho d18:0/18:0. Principal components analysis of the relative abundances of all PL class species distinguished between neuroborreliosis patients and healthy subjects. This is the first report comparing PL classes and their molecular species in neuroborreliosis patients and healthy subjects.

**Electronic supplementary material:**

The online version of this article (doi:10.1007/s11745-016-4212-3) contains supplementary material, which is available to authorized users.

## Introduction

Lyme disease is a human infection transmitted by ticks (*Ixodidae*) and caused by the spirochete *Borrelia burgdorferi*. A dramatic increase in the number of cases of Lyme disease in Europe has been reported in the past two decades (200,000 new cases/year) and the United States (15,000–20,000 new cases/year), and this number continues to rise. The most dangerous manifestation of Lyme disease is neuroborreliosis, which is associated with infection of the central nervous system. Previously, we showed enhanced phospholipid (PL) peroxidation and decreased phospholipase A_2_ (PLA_2_) activity, the main enzyme releasing peroxidation products, during neuroborreliosis [1]. Despite these findings, the pathogenesis of neuroborreliosis has still not been fully determined. However, there are indications that PL and their metabolites participate in the inflammatory response in Lyme disease [2, 3]. It is known that some PL species such as lyso-phosphatidylcholines (LysoPtdCho), phosphatidyethanolamines (PtdEtn), phosphatidylcholines (PtdCho), phosphatidylinositol (PtdIns), and sphingolipids (CerPCho) are involved in the development of inflammatory diseases such as rheumatoid arthritis, pancreatic cancer, and ovarian cancer [4–6]. To date there are no published reports that have focused on the profiles of the main PL species in plasma from patients with neuroborreliosis. This data might lead to the identification of altered metabolic pathways, and be useful for monitoring pharmacotherapy. Therefore, the aim of this study was to extend our knowledge of PL participation in the development of neuroborreliosis using a lipidomic approach.

## Materials and Methods

### Chemicals

All solvents used were of LC–MS grade. All chemicals were purchased from Sigma-Aldrich (St. Louis, MO, USA) and had greater than 95% purity. PL internal standards were purchased from Avanti Polar Lipids.

### Biological Material

We obtained plasma samples from neuroborreliosis patients and healthy subjects collected in the Department of Infectious Diseases and Neuroinfections, Medical University of Bialystok (Poland). The samples were collected from eight patients with neuroborreliosis (three female and five male) with an average age of 48 years (range 21–83). The control group consisted of eight healthy subjects (three female and five male), with an average age of 47 years (range 22–72).

The diagnosis of neuroborreliosis was confirmed by epidemiological anamnesis. Fifty percent of the neuroborreliosis patients reported previous tick bites, clinical manifestations of Bannwarth’s syndrome, lymphocytic meningitis with or without nerves paresis, and serological detection of anti-*B. burgdorferi* IgM and IgG antibodies in enzyme-linked immunosorbent assays (ELISA; *Borrelia* recombinant IgG and IgM High Sensitivity, Biomedica, Austria). In all cases, the ELISA results were confirmed by western blotting. Additionally, IgM and IgG immunoblot tests were performed (Virotech, Germany) to estimate intrathecal synthesis of antibodies in cerebrospinal fluid (CSF).

All neuroborreliosis patients had serum anti-*B. burgdorferi* antibodies, with mean titres for IgM and IgG of 24 ± 19 BBU/ml and 55 ± 23 BBU/ml, respectively. Three patients (37%) had intrathecal synthesis of IgM antibodies, five (62%) had intrathecal synthesis of IgG antibodies, and two patients (25%) had both in their CSF. Based on these criteria we diagnosed a definitive clinical picture, lymphocytary pleocytosis in CSF, intrathecal immunoglobulin synthesis and probable clinical history and at least one of the following findings: lymphocytary pleocytosis in CSF, erythema migrans >5 cm in diameter, or prompt clinical response to antibiotic treatment. In all patients tick-borne encephalitis was excluded based on serological tests on serum and CSF.

The exclusion criteria for both groups were as follows: pregnancy, lack of written consent, or recent treatment with nonsteroidal anti-inflammatory drugs, steroids, or oral contraceptives. In the control group, there was no history of other diseases which could influence increased PL oxidation, e.g., arthritis of any etiology. Patients and healthy subjects with alcohol abuse and heavy smokers were also excluded from the study. The study had approval from the Local Bioethics Committee at the Medical University of Bialystok, and written informed consent was obtained from all patients.

Blood was collected from all participants into ethylenediaminetetraacetic acid tubes and centrifuged at 2000×*g* (4 °C) to obtain the plasma.

### Lipid Extraction

Total lipids from all plasma samples were extracted using a modified Folch method [7]. In brief, 1.5 ml of ice-cold methanol was added to each 200 µl of plasma sample and vortexed thoroughly. Then, 3 ml of chloroform was added, vortexed, and incubated on ice for 60 min. To induce phase separation, 1.25 ml ultra-pure Milli-Q water was added. After 10 min incubation on ice, samples were centrifuged at 2500×*g* for 10 min at room temperature to obtain the aqueous top and organic bottom phases from which lipids were obtained.

### PL Quantification and Separation of PL Classes by Thin Layer Chromatography (TLC)

Silica gel TLC plates 20 × 20 cm (Merck, Darmstadt, Germany) were used to separate the PL classes. First, plates were treated with 2.3% boric acid in ethanol. Then, 20 µl of 20–30 µg PL extract were seeded on the TLC plate and developed using a mixture of chloroform/ethanol/water/triethylamine 35:30:7:35, v/v/v/v. PL spots were observed by exposure to primuline 50 µg/100 mL acetone:water, 80:20, v/v, and visualized with a UV lamp at *λ* = 254 nm [9]. Identification of the different PL classes was performed by comparison with PL standards applied to the same plate. Estimation of the total amount of PL in total lipid extracts and in the spots after TLC separation was performed according to Bartlett and Lewis [8]. The relative abundance (%) of each PL class was calculated by relating the amount of phosphorous in each spot to the amount of total phosphorous in each plasma lipid extract.

### PL Profile Characterization by HILIC-LC–MS

PL classes were separated by hydrophilic interaction liquid chromatography (HILIC), performed on an Ultra high performance liquid chromatography (UPLC) system (Agilent 1290; Agilent Technologies, Santa Clara, CA, USA) coupled to a quadrupole time of flight mass spectrometer (QTOF) (Agilent 6540; Agilent Technologies, Santa Clara, CA, USA). The characterization of PL classes and individual species within each class was achieved by data dependent ESI–QTOF–MS/MS in negative mode with formation of [M-H]^−^ for PtdIns and PtdEtn, and with formation of [M + OAc]^−^ for PtdCho, LysoPtdCho, and CerPCho.

Mobile phase A consisted of 25% water, 50% acetonitrile, 25% v/v methanol with 10 mM ammonium acetate. Mobile phase B consisted of acetonitrile 60%, methanol 40% with 10 mM ammonium acetate. Total PL samples (20 µg) were diluted in mobile phase B and 5 µl of the mixture was introduced into an Ascentis Si HPLC Pore column 15 cm × 1.0 mm, 3 µm (Sigma-Aldrich). The solvent gradient was programmed as follows: the gradient started with 0% of A, linearly increased to 100% over 20 min, held isocratically for 35 min, and returning to the initial conditions over 5 min. The flow rate through the column was 40 µl/min.

ESI Agilent Dual AJS ESI conditions were as follows: electrospray voltage, −3.0 kV; capillary temperature, 250 °C; sheath gas flow, 13 L/min. Parent scan spectra were acquired in the range of* m*/*z* 100–1500. Collision energy was fixed at 35 for MS/MS. Data acquisition was carried out with Mass Hunter data software version B0.6.0 (Agilent Technologies, Santa Clara, CA, USA). An isolation width of ~1.3 Da was used for the MS/MS experiments. MS/MS was performed for each ion to identify and confirm their structure, according to the typical fragmentation pathways [10]. Internal standards PtdCho 14:0/14:0, PtdIns 16:0/16:0, and PE 14:0/14:0, (Avanti Polar Lipids) were used to confirm the ion variations observed in the MS spectra according to Lipid Maps [11]. The relative abundance of each ion was calculated by normalizing the area of each extracted ion chromatogram peak to the area of an internal standard.

### Statistical Analysis

Means ± standard deviations (SD) were calculated for all data. The relative ion abundances obtained by HILIC-LC–MS from the two groups of plasma extracts were analyzed using one-way analysis of variance (ANOVA) with Bonferroni post hoc tests used to determine significant differences between samples. Differences were considered significant if *p* < 0.05. Statistical analysis was performed using GraphPad Prism 5 for Windows version 5.0.1 (GraphPad Software, San Diego, CA, USA). Principal component analysis (PCA) classification of the data was performed using SIMCA-P + version 12.0.1 software (Umetrics, Umeå, Sweden). This was performed using data from the most abundant PL species in each class, following log transformation to achieve normal distribution of the data, followed by Pareto-scaling.

## Results and Discussion

**Fig. 1:**
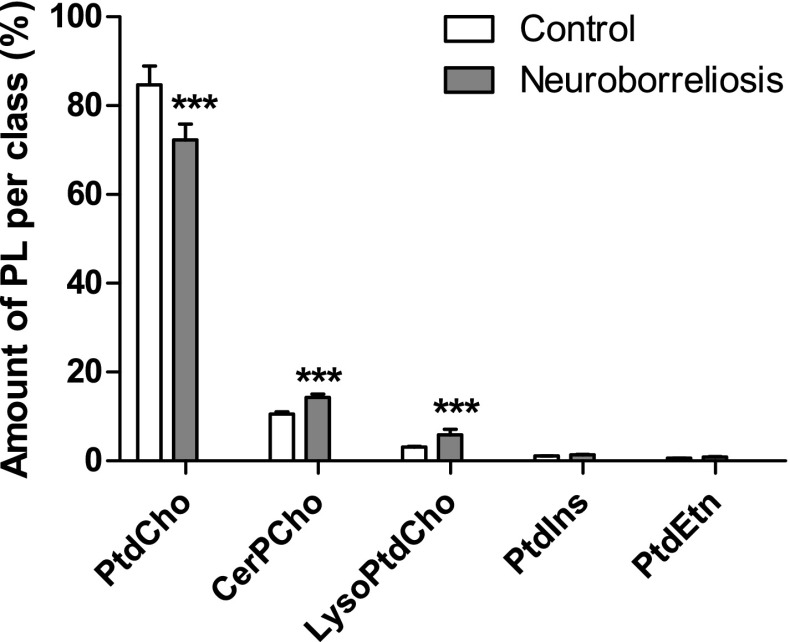
Amount of plasma phospholipid per class in healthy subjects and neuroborreliosis patients. Data obtained by colorimetric assay preformed after TLC separation (values are mean ± SD ***p < 0.001)

The pathology of many infectious diseases, including Lyme disease, is associated with altered PL metabolism. The composition of PL can be considered an index of the organism in health and disease, as well as an indicator of metabolic responses to pharmacotherapy [12]. TLC analysis of plasma extracts confirmed that the most abundant PL class in all plasma samples was PtdCho, while CerPCho, LysoPtdCho, PtdIns, and PtdEtn were less abundant (Fig. 1), which is the typical PL pattern for human plasma [13]. The results of PL quantification by phosphorous assay showed decreases of PtdCho in the plasma of neuroborreliosis patients compared with controls, while CerPCho and LysoPtdCho were significantly more abundant (Fig. 1).

The formation of LysoPtdCho species can occur as a result of oxidative degradation of PtdCho, as demonstrated by an increase of lipid peroxidation in plasma of neuroborreliosis patients in our previous work [1]. The decrease in plasma PtdCho may explain the increase in lipid peroxidation found in those patients. The other PL classes (PtdIns and PtdEtn) were not significantly different in healthy subjects and patients. We then analyzed PL profiles using a HILIC-LC MS/MS approach, focusing on the four main classes: phosphatidylcholine, sphingomyelin, lysophosphatidylcholine, and phosphatidylethanolamine and phosphatidylinositol.

### Phosphatidylcholine

**Fig. 2:**
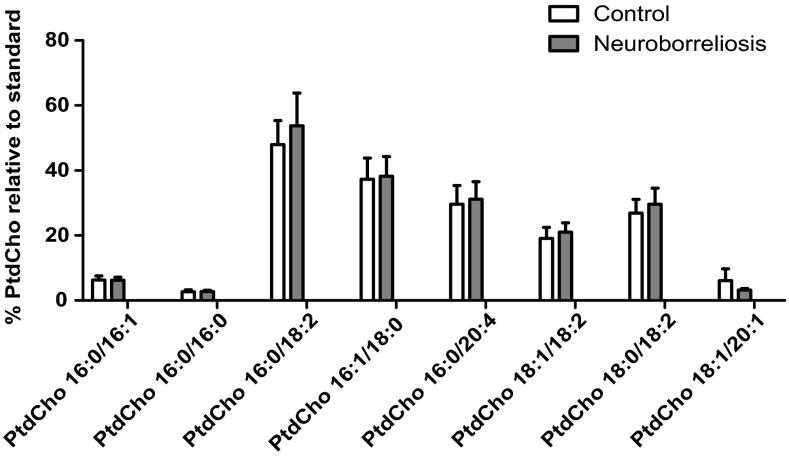
PtdCho molecular species relative compositions in healthy subjects and neuroborreliosis patients (values are mean ± SD). The PtdCho species are PtdCho 16:0/16:1, PtdCho 16:0/16:0, PtdCho 16:0/18:2, PtdCho 16:1/18:0, PtdCho 16:0/20:4, PtdCho 18:1/18:2, PtdCho 18:0/18:2, and PtdCho 18:1/20:1, corresponding to [M + OAc]^−^ at* m*/*z* 790.4854, 792.5748, 816.5764, 818.5904, 840.5762, 842.5906, 844.6069, and 872.6947, respectively

The most abundant PtdCho species observed in plasma [13] were identified by HILIC-LC–MS, and the fatty acyl chain compositions annotated. No significant differences in the relative abundance of PtdCho species were found between healthy subjects and neuroborreliosis patients (Fig. 2).

### Sphingomyelin

**Fig. 3:**
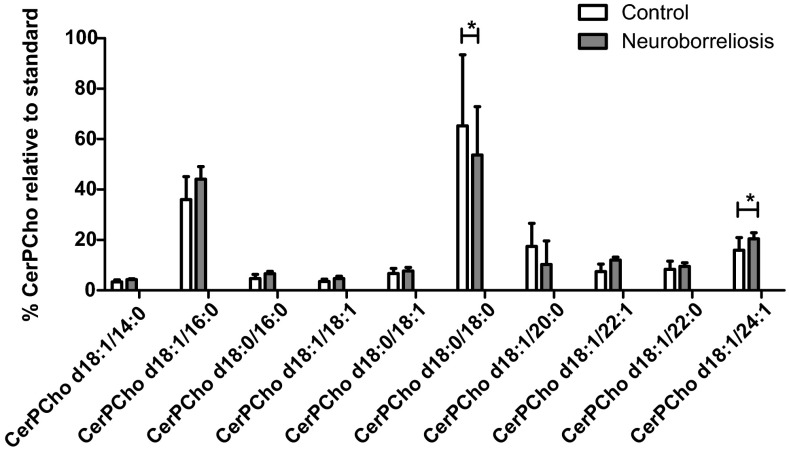
CerPCho molecular species relative compositions in plasma of healthy subjects and neuroborreliosis patients (values are mean ± SD, *p < 0.05). The CerPCho species are CerPCho d18:1/14:0, CerPCho d18:1/16:0, CerPCho d18:0/16:0, CerPCho d18:1/18:1, CerPCho d18:0/18:1, CerPCho d18:1/18:0, CerPCho d18:0/18:0, CerPCho d18:1/20:0, CerPCho d18:1/22:1, CerPCho d18:1/22:0, and CerPCho d18:1/24:1, observed as [M + OAc]^−^ ions at* m*/*z* 733.5508, 761.5823, 763.5879, 787.5974, 789.6128, 791.4909, 817.6433, 843.5654, 845.6746, and 871.6906, respectively

The sphingomyelin profiles of all plasma extracts examined showed three major species, CerPCho d18:1/16:0, CerPCho d18:0/18:0, and CerPCho d18:1/24:1, observed as [M + OAc]^−^ ions at *m*/*z* 761.5823, 791.4909, and 871.6906, respectively (Fig. 3). LC–MS data analysis showed an increase in the relative abundance of CerPCho d18:1/24:1 and a decrease in the relative abundance of CerPCho d18:0/18:0 (Fig. 3).

These results suggest that CerPCho may play an important role in the development of pathological changes in the central nervous systems of neuroborreliosis patients. It has been shown that *B. burgdorferi* can induce an autoimmune attack on myelin sheaths, as glycolipid galactocerebroside, the major component of myelin, has structural similarities to the *B. burgdorferi* glycolipid antigen BbGL-2 [14]. The inflammatory demyelination of neurons, as well as peripheral nerves, in Lyme disease has been suggested in previous studies [15, 16]. We analyzed the relative abundances of major CerPCho species using PCA. There were some differences in the clustering of samples from healthy volunteers and neuroborreliosis patients (Fig.S1 supplementary material), but these groups did not form distinct clusters.

### Lysophosphatidylcholine

**Fig. 4:**
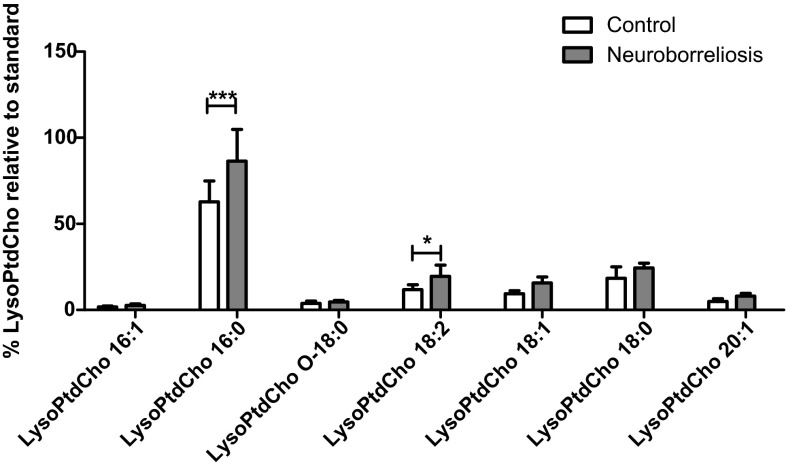
LysoPtdCho molecular species relative abundances in plasma of healthy subjects and neuroborreliosis patients (values are mean ± SD *p < 0.05 and ***p < 0.001). The LysoPtdCho species are LysoPtdCho 16:1, LysoPtdCho 16:0, LysoPtdCho O-18:0, LysoPtdCho 18:2, LysoPtdCho 18:1, LysoPtdCho 18:0 and LysoPtdCho 20:1, corresponding to [M + OAc]^−^ at* m*/*z* 552.331, 554.3472, 568.3603, 578.3470, 580.3621, 582.3781, and 608.3189, respectively

All of the most abundant LysoPtdCho species identified showed the tendency to increase in patients, with LysoPtdCho 16:0 and LysoPtdCho 18:2 significantly more abundant in neuroborreliosis patients than in controls (Fig. 4).

It is well known that LysoPtdCho can be generated under physiological conditions by PLA_2_-mediated hydrolysis of PtdCho [17], or from the hydrolysis of oxidized PtdCho by PAF-acetylhydrolase [18]. Both mechanisms are possible, although previous work demonstrating increased lipid peroxidation in neuroborreliosis patients suggests that the latter mechanism may prevail [1]. LysoPtdCho are probably involved in demyelination [19], although, to date no substantiated data supporting LysoPtdCho-induced demyelination in Lyme disease has been published. PCA analysis of the major LysoPtdCho species completely distinguished between neuroborreliosis patients and healthy controls (Fig. S2, Supplementary Material).

### Phosphatidylethanolamine and Phosphatidylinositol

**Fig. 5:**
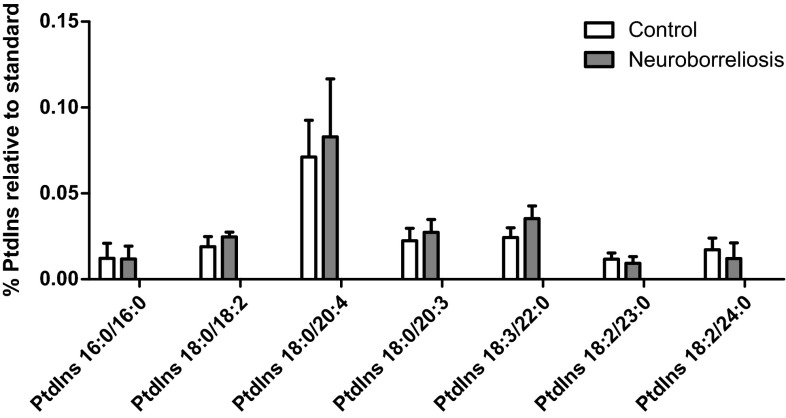
The most abundant PtdIns molecular species relative abundances in plasma of healthy subjects, neuroborreliosis and Lyme arthritis patients (values are mean ± SD). The PtdIns species are PtdIns 16:0/16:0, PtdIns 18:0/18:2, PtdIns 18:0/20:4, PtdIns 18:0/20:3, PtdIns 18:3/22:0, PtdIns 18:2/23:0 and PtdIns 18:2/24:0, corresponding to [M-H]^−^ at* m*/*z* 809.5136, 861.5501, 885.5502, 887.5612, 915.5995, 931.5954, and 945.61127, respectively

**Fig. 6:**
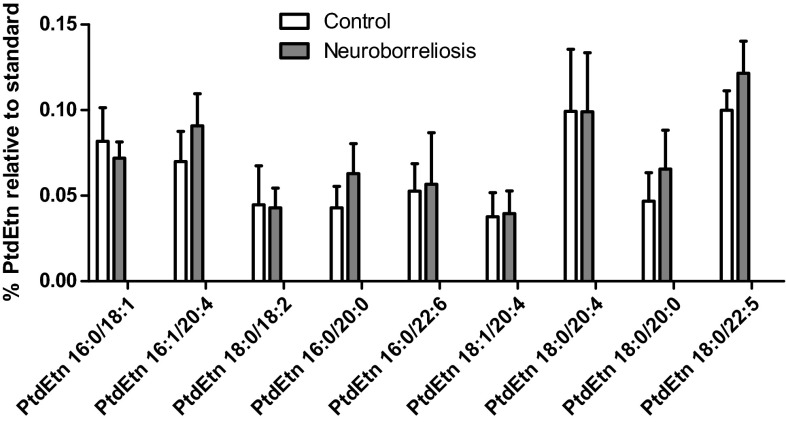
The most abundant PtdEtn molecular species relative abundances in plasma of healthy subjects, neuroborreliosis, and Lyme arthritis patients (values are mean ± SD). The PtdEtn species are PtdEtn 16:0/18:1, PtdEtn 16:1/20:4, PtdEtn 18:0/18:2, PtdEtn 16:0/20:0, PtdEtn 16:0/22:6, PtdEtn 18:1/20:4, PtdEtn 18:0/20:4, PtdEtn 18:0/20:0, and PtdEtn 18:0/22:5, corresponding to [M-H]^−^ at 716.4592, 738.5203, 742.5389, 746.5134, 762.5092, 764.5230, 766.5394, 774.5443, and 792.4936, respectively

PtdIns and PtdEtn (Figs. 5 and 6) were not significantly different between healthy subjects and neuroborreliosis patients.

Finally, we analyzed all the PL classes (PtdCho, LysoPtdCho, CerPCho, PtdEtn, and PtdIns) using PCA. The resulting plot (Fig. S3, Supplementary Material) revealed a good separation between healthy subjects and neuroborreliosis patients. This indicates that the plasma PL profiles of patients is significantly different from those of the controls.

In conclusion, to our knowledge, this is the first report of differences in plasma PL classes and their molecular species in neuroborreliosis patients and healthy subjects. Total PL quantification showed that the abundance of PtdCho in plasma is significantly lower in neuroborreliosis patients than in controls. However, the abundances of CerPCho and LysoPtdCho were significantly higher in these patients. HILIC-LC–MS data showed that the two most abundant lysophosphatidylcholines, LysoPtdCho 16:0 and LysoPtdCho 18:2, were significantly different between neuroborreliosis and controls; although, the relevance of this finding remains to be determined. Moreover, significant differences in the molecular composition of sphingomyelin profiles were also observed. The plasma of neuroborreliosis patients had a significantly higher relative abundance of CerPCho d18:1/24:1 and a lower relative abundance of CerPCho d18:0/18:0. These changes could be related to the evolution of the disease, including the occurrence of the demyelination process. PCA revealed a good separation of the relative abundances of all PL class species in healthy controls and neuroborreliosis patients with PC axes revealing almost complete distinction between them. Further studies are needed to clarify if these changes are specific to neuroborreliosis, and whether and how they are related to its pathogenesis. These results may be a useful starting point in defining potential PL neuroborreliosis biomarkers.

## Electronic supplementary material

Below is the link to the electronic supplementary material.

Supplementary material 1 (DOC 138 kb)

## Abbreviations

CerPCho: Sphingomyelin
ESI: Electrospray ionization
HILIC: Hydrophilic interaction liquid chromatography
LysoPtdCho: Lysophosphatidylcholine
PAF: Platelet-activating factor
PCA: Principal component analysis
PLA_2_: Phospholipase A_2_
PtdCho: Phosphatidylcholine
PtdEtn: Phosphatidylethanolamine
PtdIns: Phosphatidylinositol
QTOF: Quadrupole time of flight mass spectrometer
TLC: Thin layer chromatography
UPLC: Ultra high performance liquid chromatography
