# Supplementary material for: Phospholipidomic Analysis Reveals Changes in Sphingomyelin and Lysophosphatidylcholine Profiles in Plasma from Patients with Neuroborreliosis
Source: Lipids. 2016 Nov 10;52(1):93–8. doi: 10.1007/s11745-016-4212-3 (PMC5222908; doi:10.1007/s11745-016-4212-3)
Supplement: Supplementary file 1 — Supplementary material 1 (DOC 138 kb) [file 11745_2016_4212_MOESM1_ESM.doc]

**
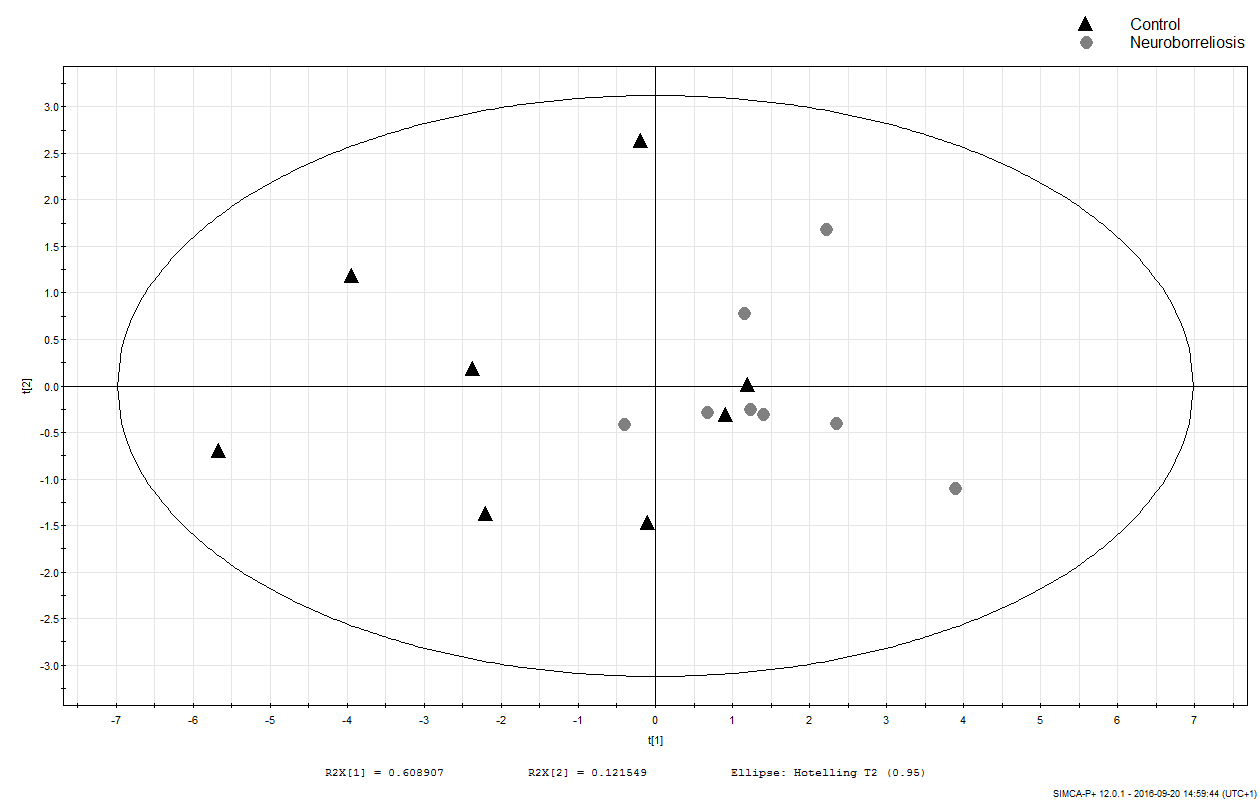
**

**Fig. S1** PCA plot of CerPCho species relative abundances (calculated as the ratio of each species’ peak area to the peak area of ISTD taken from EIC) determined by HILIC-LC-MS in healthy and neuroborreliosis subjects. Black triangles indicate healthy volunteers, while the grey dots represent neuroborreliosis patients.

**
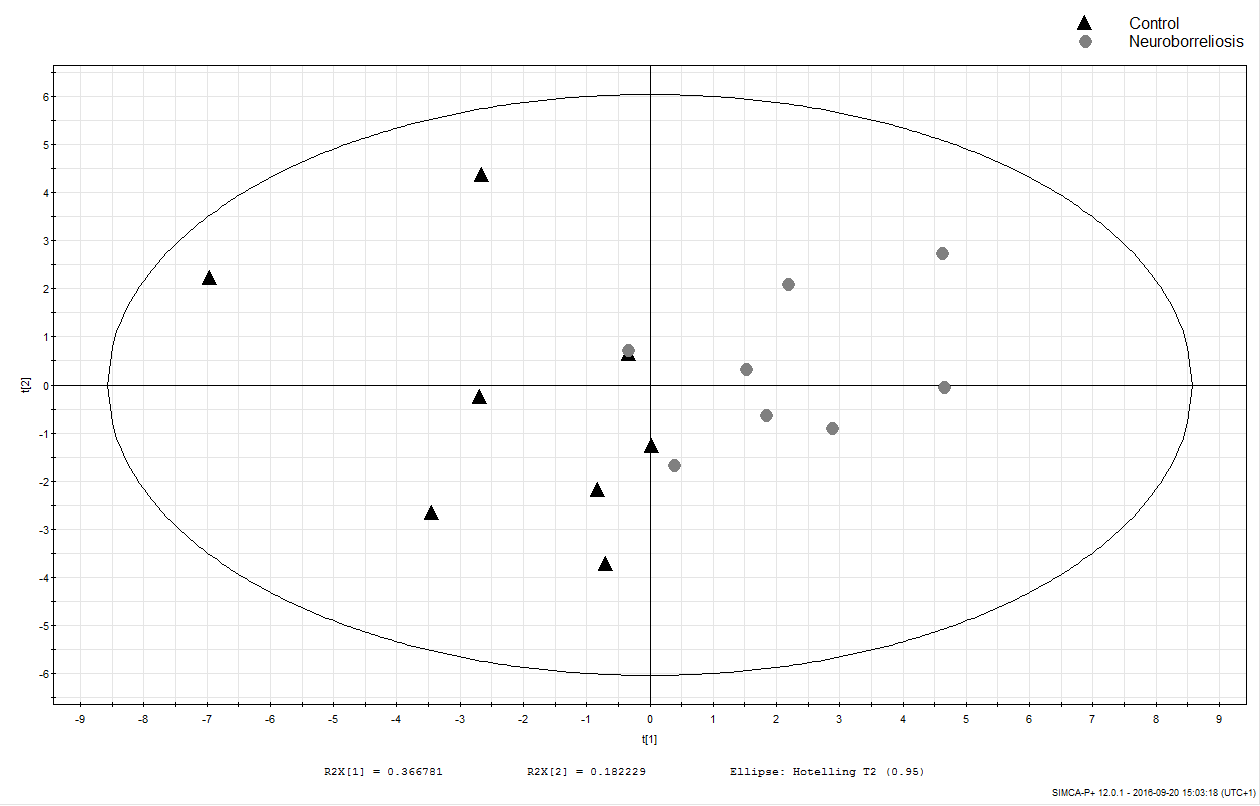
**

**Fig. S2** PCA plot of LysoPtdCho species relative abundances (calculated as the ratio of each species’ peak area to the peak area of ISTD taken from EIC) determined by HILIC-LC-MS in healthy and neuroborreliosis subjects. Black triangles indicate healthy volunteers, while the grey dots represent neuroborreliosis patients.

**
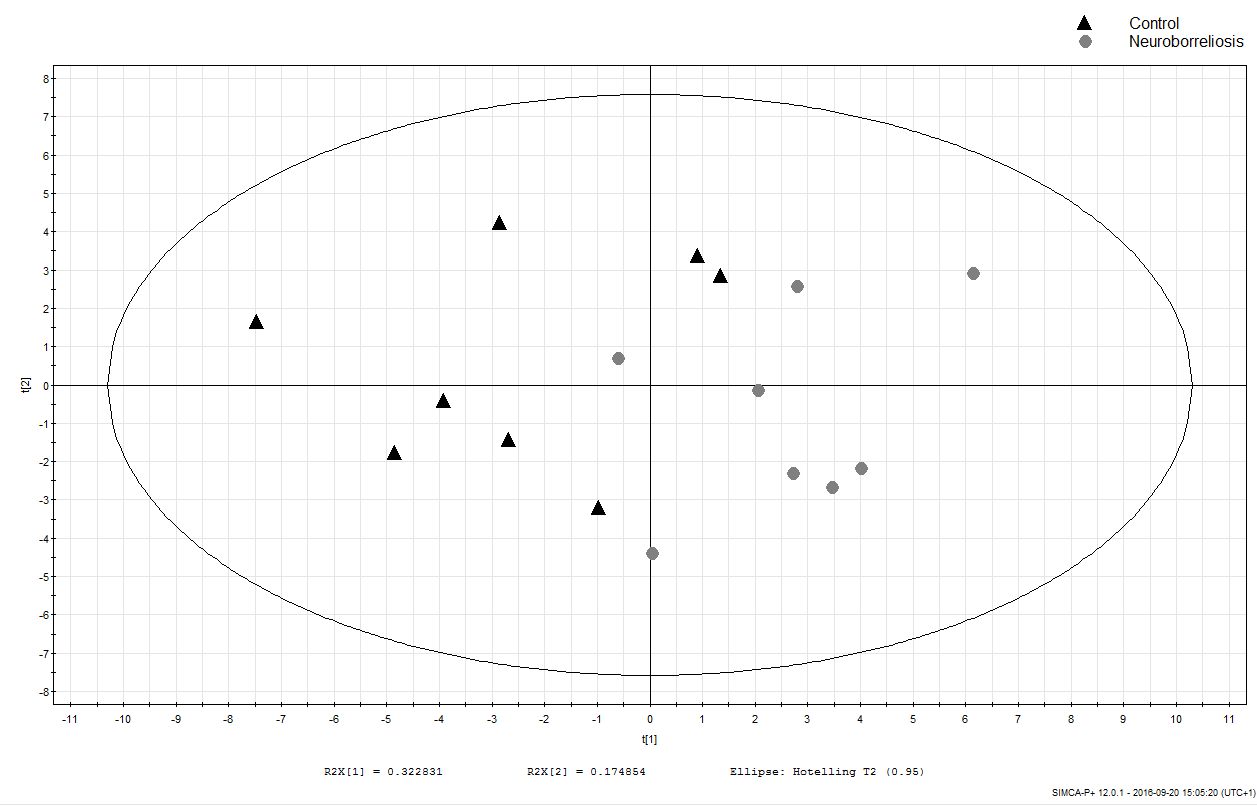
**

**Fig. S3** PCA plot of the relative abundances of all phospholipid class species (calculated as the ratio of each species’ peak area to the peak area of ISTD taken from EIC) determined by HILIC-LC-MS in healthy and neuroborreliosis subjects. Black triangles indicate healthy volunteers, while the grey dots represent neuroborreliosis patients.
